# Oral Angiolymphoid Hyperplasia With Eosinophilia Exhibiting Cutaneous‐Type Histopathologic Features: Clinical Regression Following Hormonal Withdrawal and a 50‐Year Review

**DOI:** 10.1111/cup.70072

**Published:** 2026-02-24

**Authors:** Pedro Vinícius Santos de Jesus, Louise Cristina Santos, Gilberth Tadeu Dos Santos Aciole, John Lennon Silva Cunha, Nicole Lonni, Matheus Antoni da Silva Costa, Elena Riet Correa Rivero, Rogério Gondak, Ricardo Luíz Cavalcanti de Albuquerque‐Júnior

**Affiliations:** ^1^ School of Dentistry Tiradentes University Aracaju Sergipe Brazil; ^2^ Brazilian Army Aracaju Sergipe Brazil; ^3^ Center for Biological and Health Sciences Federal University of Western Bahia Barreiros Bahia Brazil; ^4^ Center for Health Sciences, Postgraduate Program in Dentistry Federal University of Santa Catarina Florianópolis Santa Catarina Brazil; ^5^ Department of Pathology, Postgraduate Program in Dentistry Federal University of Santa Catarina Florianópolis Santa Catarina Brazil

**Keywords:** angiolymphoid hyperplasia with eosinophilia, contraceptive drugs, differential diagnosis, oral mucosa, periodontal disease

## Abstract

Angiolymphoid hyperplasia with eosinophilia (ALHE) is a rare benign vascular proliferation, typically affecting the head and neck's subcutaneous tissue, with infrequent involvement of the oral mucosa. We report the case of a 30‐year‐old female patient on oral contraceptives who presented with a hyperplastic, reddish‐purple, asymptomatic, and bleeding gingival lesion in the left maxillary gingiva. Clinical examination revealed a 10 mm periodontal pocket in the upper left permanent canine, with horizontal bone loss affecting the adjacent lateral incisor and canine. Blood analysis showed mild eosinophilia. An initial incisional biopsy suggested a chronic inflammatory process, but due to persistent lesion growth, histopathological revaluation confirmed ALHE. Management included contraceptive discontinuation, scaling and root planing, and intralesional corticosteroid therapy, resulting in complete remission within 14 days. This case highlights the importance of considering ALHE in the differential diagnosis of hyperplastic gingival lesions with periodontal involvement. Furthermore, conservative treatment may offer an effective alternative to surgical excision for intraoral ALHE. A review of cases of oral ALHE in the last 50 years is also provided.

## Graphical abstract

Case report of a 30‐year‐old female. Asymptomatic, red‐purple hyperplastic lesion extending from teeth 21 to 25, with bleeding upon manipulation. Differential diagnosis included: inflammatory gingival hyperplasia, plasma cell gingivitis, leukemia, and Wegener's granulomatosis. Histological examination confirmed angiolymphoid hyperplasia with eosinophilia based on vascular proliferation, lymphoid follicles, and eosinophil infiltration. In this case, lesion regression followed contraceptive discontinuation and subgingival scaling, with intralesional triamcinolone acetonide providing sustained remission for over 3 years.

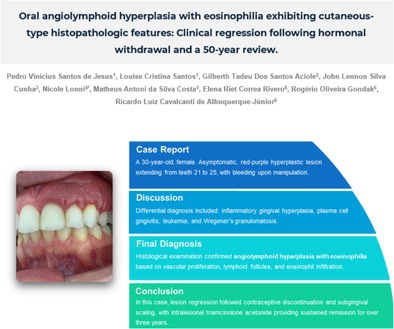

## 1 Introduction

Angiolymphoid hyperplasia with eosinophilia (ALHE) is a rare benign inflammatory vascular lesion [1]. Although the etiology of this condition is still uncertain, some studies suggest an association with trauma [2], human polyomavirus 6 infection [3], hormonal changes during pregnancy [4], and use of contraceptive drugs [5].

Clinically, ALHE presents one or more reddish‐brown papules or nodules with sudden onset and slow growth, typically in the subcutaneous tissue of the head and neck [1]. It is often asymptomatic but may cause pruritus, pain, or spontaneous bleeding. Peak incidence occurs between the third and fifth decades of life [6]. Although rare in the oral mucosa, when ALHE occurs in this anatomic site, the lips, jugal mucosa, and tongue are more affected [7, 8, 9]. The histopathological features comprise immature vessels lined by epithelioid endothelial cells, with a prominent lymphocytic and eosinophilic infiltrate [10]. Lesions are well‐circumscribed, often lobulated, and may contain lymphoid follicles with germinal centers [11].

Treatment typically involves conservative surgical excision [12]. Other more conservative options include pulsed dye laser [13], carbon dioxide laser [14], cryotherapy [15], and pharmacological approaches such as β‐blockers [16] and corticosteroids [17]. Recurrence occurs in about one‐third of cases [18].

This paper reports an atypical case of ALHE successfully treated with intralesional corticosteroid therapy and provides a review of oral ALHE cases, emphasizing differential diagnosis, treatment, and prognosis.

## 2 Case Report

**FIGURE 1:**
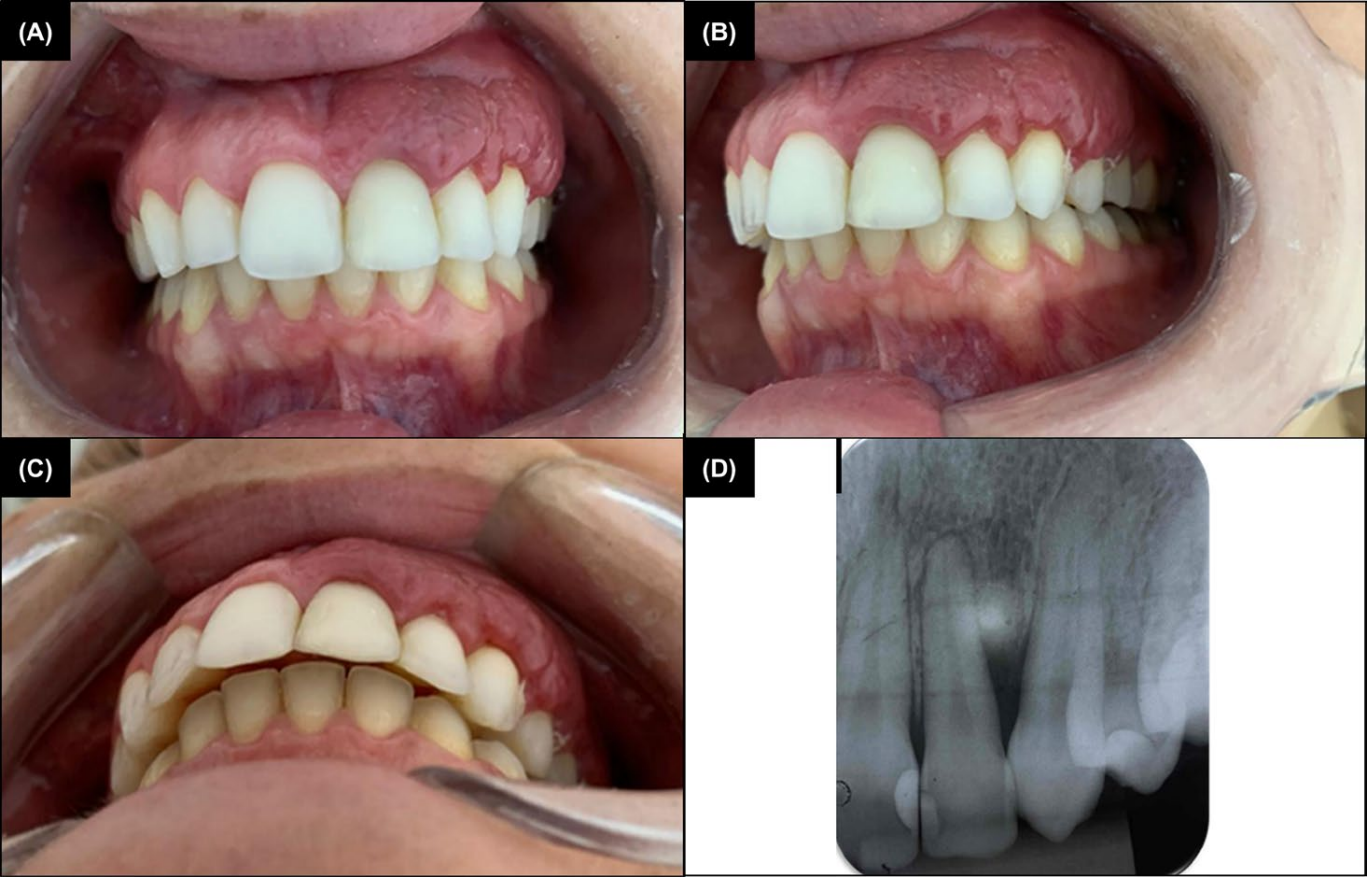
Angiolymphoid hyperplasia with eosinophilia (ALHE). Clinical aspect of the lesion in a frontal (A) and lateral (B) intraoral view, showing red‐purple periodontal hyperplasia with a rough surface, located in the region from the upper left central incisor to the upper left first premolar. (C) Bottom view showing increased periodontal volume caused by the lesion. (D) A periapical radiograph revealing severe horizontal bone loss between the upper left lateral incisor and the upper left canine.

A 30‐year‐old female patient had been using oral contraceptives (2 mg cyproterone, 0.035 mg ethinyl estradiol) for 5 years. Intraoral examination revealed an asymptomatic, red‐purple hyperplastic lesion on the left maxillary gingiva, extending from the left central incisor to the left first premolar, with bleeding upon manipulation. A 10 mm periodontal pocket was detected at the maxillary left canine, and radiography showed severe horizontal bone loss between the maxillary left lateral incisor and canine (Figure 1). Peripheral blood analysis revealed mild eosinophilia, while other hematologic values, including total leukocyte and platelet counts, were within normal limits. Initial differential diagnoses included inflammatory gingival hyperplasia and granulomatosis with polyangiitis.

**FIGURE 2:**
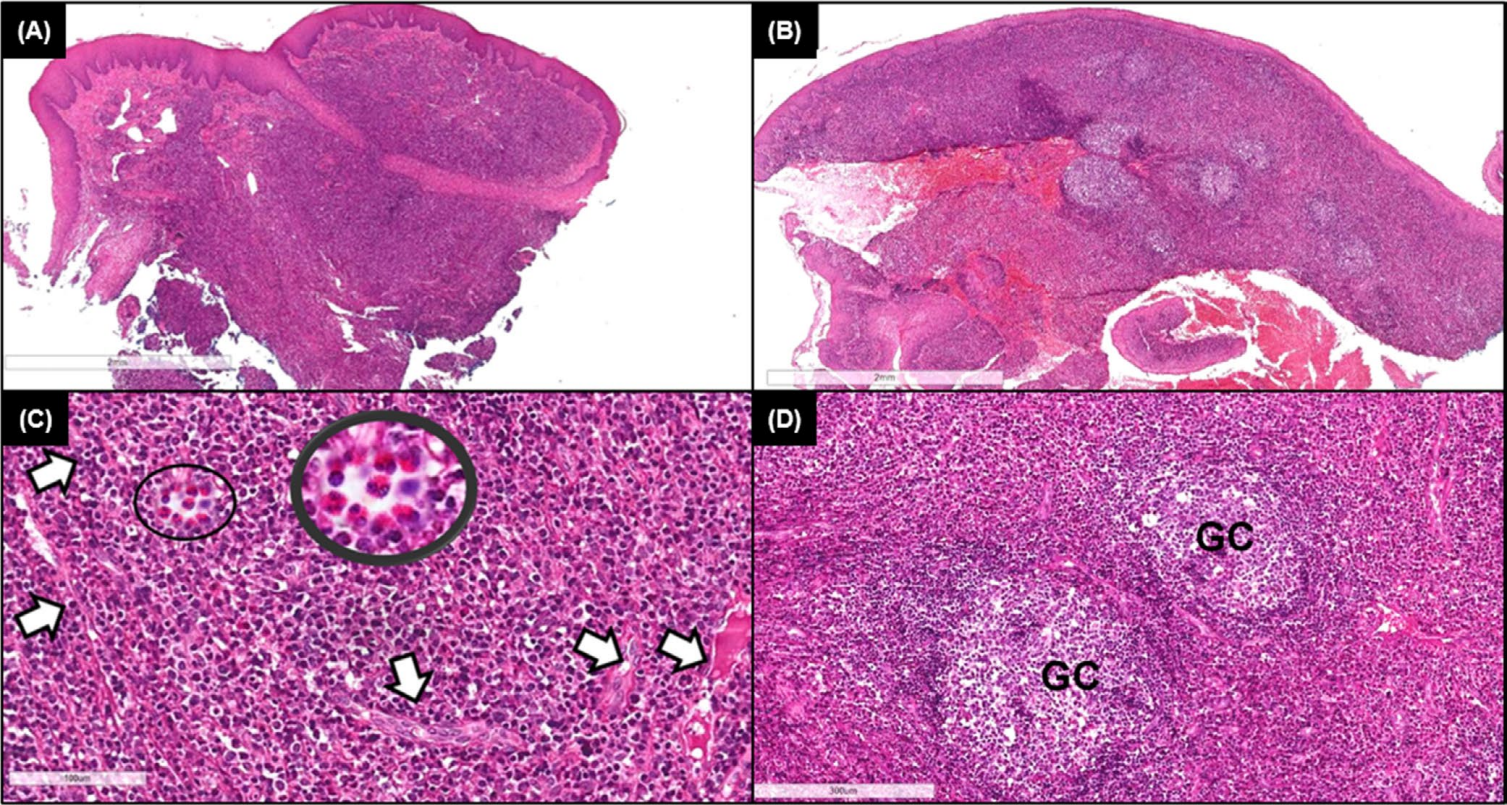
Angiolymphoid hyperplasia with eosinophilia (ALHE). Photomicrographs of H&E stained histological sections obtained from the incisional biopsy of the paraffin‐embedded specimen. (A, B) Panoramic view showing oral mucosa with a slightly lobulated surface lined by hyperplastic and acanthotic squamous epithelium and lamina propria intensely infiltrated by inflammatory cells (×40). (C) A dense sheet of inflammatory cells permeated by narrowed blood vessels lined by epithelioid endothelial cells (white arrows) (×200). In detailed magnification (×400), numerous eosinophils (black circles) are observed permeating mononuclear inflammatory cells and neoformed vessels. (D) Areas of well‐formed lymphoid follicles with well‐developed germinal centers (GC) (×100).

Oral contraceptives were temporarily discontinued, and an incisional biopsy revealed a chronic inflammatory process. Despite significant improvement following scaling and root planing, the lesion persisted. Due to the nonspecific findings in the initial histopathological report, a second review was conducted, revealing capillary proliferation with epithelioid endothelial cells, dense lymphoplasmacytic infiltration with eosinophils, and focal lymphoid follicle formation (Figure 2). These findings led to the diagnosis of ALHE.

**FIGURE 3:**
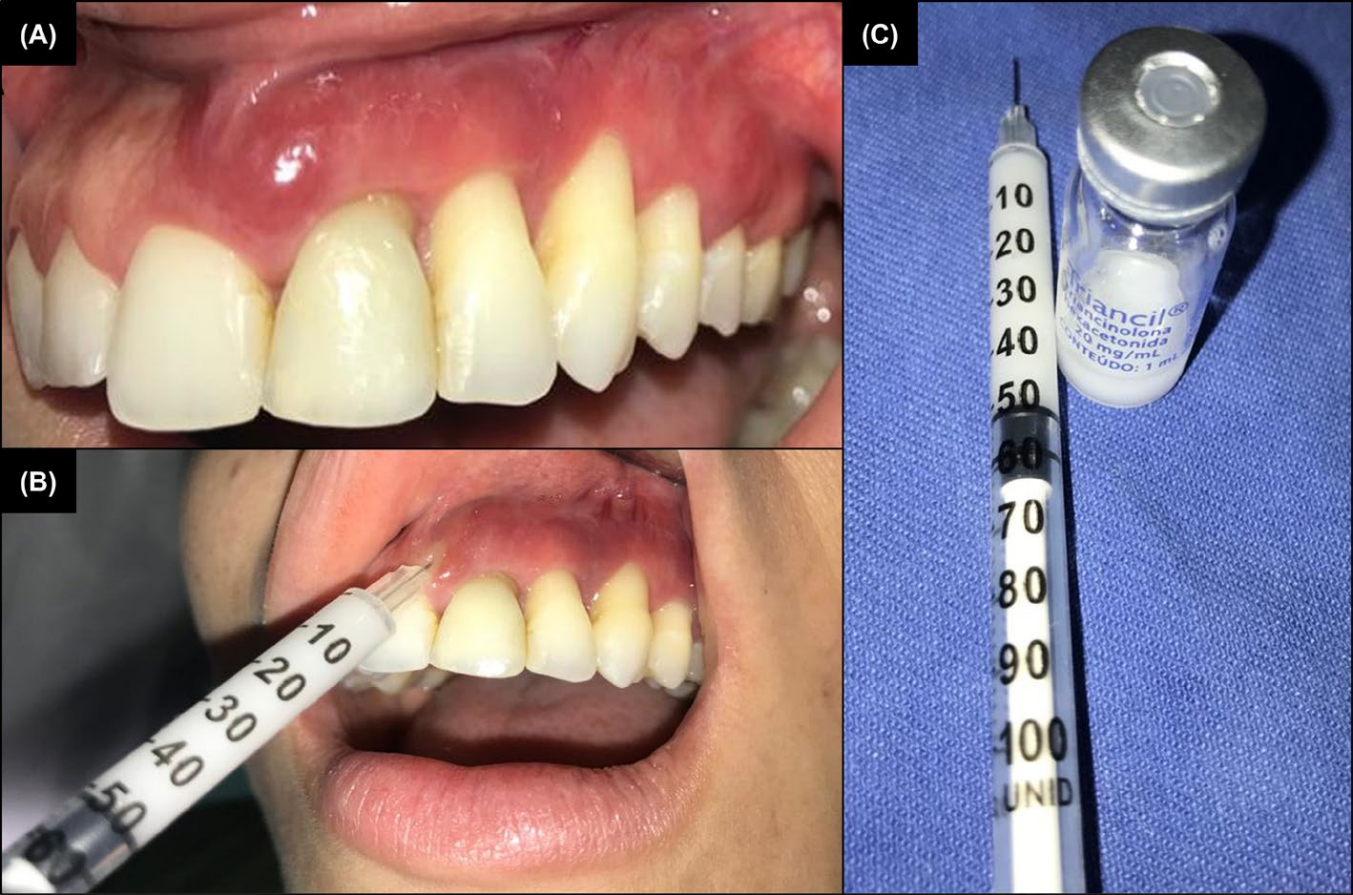
Angiolymphoid hyperplasia with eosinophilia (ALHE). (A) Clinical appearance upon return after the first 2 weeks, with periodontal treatment only (scaling and root planing). (B) Triamcinolone acetonide prepared for infusion. (C) Infusion of triamcinolone acetonide into the residual lesion.

**FIGURE 4:**
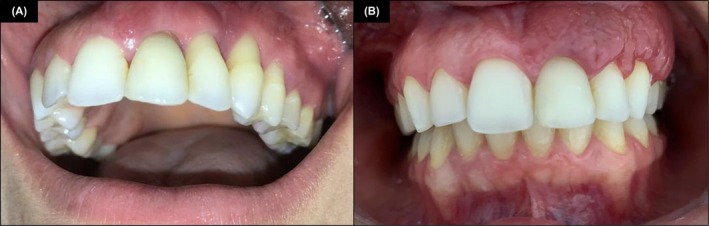
Angiolymphoid hyperplasia with eosinophilia (ALHE). (A) Clinical presentation of the lesion at the initial consultation with the dental surgeon. (B) Complete remission of the lesion and restored periodontal health following conservative treatment with scaling, root planing, and intralesional triamcinolone acetonide.

Given the partial response to conservative treatment, intralesional triamcinolone acetonide (0.5 mL, 20 mg/mL) was administered, leading to complete remission within 2 weeks (Figure 3). The patient remains recurrence‐free after 3 years of follow‐up (Figure 4). Informed consent was obtained from the participant, who was fully informed about the purpose and details of the case report and provided written authorization for the inclusion of their personal and medical information.

## 3 Discussion

ALHE was first described by Wells and Whimster in 1969 as a vasculoproliferative lesion with a dense inflammatory infiltrate rich in lymphocytes and eosinophils [19]. Despite extensive investigation, its precise etiology remains unclear. The predominant hypothesis suggests ALHE as a hyperplastic reparative vascular response to trauma [1], while an alternative theory proposes a lymphoproliferative origin [20].

A review of 57 oral ALHE cases (Table 1), including the present one, showed a slight male predominance (1.54:1) and an age range of 3–82 years (mean 38.4), consistent with broader series [12]. Oral manifestations remain rare [50, 51], most commonly affecting the lip (44.6%), tongue (28.6%), and buccal mucosa (12.5%), with nodular lesions predominating (76.8%). Gingival involvement is exceedingly uncommon, with only two prior cases—one with bone loss and one without. These epidemiologic and clinical trends indicate that the present case deviates from the typical profile in three important ways: (1) its gingival location, (2) its periodontal bone loss mimicking inflammatory disease, and (3) its apparent hormonal responsiveness in a female patient.

**TABLE 1:** Summary of previously reported clinical data on ALHE in the oral mucosa.

Age (years)	Sex	Lesion	Location	Clinical diagnosis	Peripheral eosinophilia	References
37	F	Nodule	Lower lip	Kaposi's sarcoma	NR	Rosai and Akerman [21]
28	F	Macula	Palate	Lymphoma	Present	Saxe and Kahn [22]
22	M	Nodule	Upper/lower lip	NR	NR	Dickens [23]
28	M	Nodule	Upper lip	Salivary gland adenoma	NR	Buckerffeld and Edwards [24]
46	M	Nodule	Upper lip	NR	NR	Eveson and Lucas [25]
13	M	Edema	Lower lip	NR	Absent	Buchner et al. [26]
28	M	Nodule	Tongue	Pyogenic granuloma	Absent	Massa et al. [27]
32	M	Nodule	Upper lip	NR	Absent	Peters et al. [28]
31	M	Macula	Tongue	NR	Absent	Iguchi et al. [29]
25	F	Nodule	Palate	NR	NR	Moran et al. [30]
42	F	Nodule	Buccal mucosa	NR	NR	Kabani et al. [31]
82	M	Ulcer	Tongue	Epidermoid carcinoma	NR	Razquin et al. [32]
48	M	Nodule	Tongue	NR	NR	Artazkoz del Toro et al. [33]
55	M	Ulcer	Lower lip	Epidermoid carcinoma	NR	López and Battaglino [34]
17	M	Nodule	Buccal mucosa	Pyogenic granuloma	NR	Toeg et al. [35]
12	M	Ulcer	Buccal mucosa	NR	NR	Toeg et al. [35]
65	M	Nodule	Upper lip	Epithelioid hemangioma	NR	Renshaw and Rosai [36]
43	F	Nodule	Upper lip	Epithelioid hemangioma	NR	Renshaw and Rosai [36]
3	M	Nodule	Upper lip	Epithelioid hemangioma	NR	Renshaw and Rosai [36]
34	F	Pruritus	Lower lip	Epithelioid hemangioma	NR	Renshaw and Rosai [36]
59	M	Nodule	Buccal mucosa	NR	Absent	Misselevich et al. [37]
30	F	Nodule	Upper lip	NR	Present	Bartralot et al. [38]
27	F	Nodule	Buccal mucosa	NR	NR	Martin‐Granizo et al. [39]
54	M	Nodule	Lower lip	NR	NR	Martin‐Granizo et al. [39]
43	F	Nodule	Tongue	NR	NR	Martin‐Granizo et al. [39]
30	F	Nodule	Upper lip	NR	Absent	Mariatos et al. [40]
23	M	Ulcer	Tongue	Malignant tumor	NR	Shimoyama et al. [41]
60	M	Nodule	Buccal mucosa	NR	Present	Tsuboi et al. [42]
56	M	Ulcer	Tongue	NR	Present	Park et al. [43]
40	M	Nodule	Upper lip	NR	Present	Suzuki et al. [44]
59	M	Nodule	Tongue/Vallecula	Hemangioma or rhabdomyoma	NR	Jacob et al. [45]
42	M	Nodule	Palate	Salivary gland adenoma	NR	Sun et al. [46]
48	M	Nodule	Tongue	Vascular tumor	NR	Sun et al. [46]
52	M	Nodule	Tongue	Vascular tumor	NR	Sun et al. [46]
65	F	Nodule	Tongue	Pyogenic granuloma	NR	Sun et al. [46]
31	F	Nodule	Upper lip	Vascular tumor	NR	Sun et al. [46]
8	M	Nodule	Lower lip	Pyogenic granuloma	NR	Sun et al. [46]
32	F	Ulcer	Tongue	Epidermoid carcinoma	NR	Sun et al. [46]
36	F	Nodule	Upper lip	Pleomorphic adenoma	Absent	Salinas et al. [47]
75	F	Ulcer	Tongue	NR	Absent	Garrido‐Ríos et al. [48]
65	F	Nodule	Lower lip	NR	Present	Miteva et al. [49]
25	F	Nodule	Upper and lower lip	Atypical granuloma	Absent	Aggarwal and Keluskar [50]
30	M	Nodule	Gingiva	NR	Absent	Kumar et al. [51]
52	M	Nodule	Buccal mucosa	Lipoma	Absent	Henriques et al. [52]
50	M	Nodule	Upper lip	Salivary gland neoplasia	Absent	Tenório et al. [53]
7	M	Nodule	Lower lip	Pyogenic granuloma	Absent	Venkatesan and Singh [54]
30	M	Nodule	Upper lip	Benign salivary gland neoplasm	NR	Mathew et al. [55]
58	M	Nodule	Tongue	NR	NR	Lin et al. [7]
32	F	Nodule	Gingiva	NR	Present	Tovío‐Martínez et al. [56]
60	F	Nodule	Tongue	NR	NR	Volpe et al. [57]
37	M	Multiple nodules	Upper lip	NR	Present	Brahs et al. [8]
10	F	Nodule	Lower lip	NR	Absent	Chaturvedi et al. [58]
52	F	Nodule	Gingiva	NR	Absent	Assis et al. [59]
0.5	M	Papule	Tongue	NR	Absent	Ansari et al. [9]
30	M	Nodule	Tongue	NR	NR	Prabakaran et al. [60]
60	F	Ulcer	Tongue	NR	NR	Mohanty et al. [61]
30	F	Hyperplasia	Gingiva	Idiopathic gingival hyperplasia	Present	Current case

Abbreviations: F, female; M, male; NR, not reported.

The clinical differential diagnosis encompassed inflammatory gingival hyperplasia, plasma cell gingivitis, leukemia, eosinophilic granulomatosis with polyangiitis (EGPA, formerly Churg–Strauss syndrome), and lobular capillary hemangioma (pyogenic granuloma, PG). Inflammatory gingival hyperplasia is commonly associated with periodontal disease or drug‐induced gingival overgrowth [50]. Plasma cell gingivitis, a hypersensitivity reaction to various antigens, manifests as marked gingival inflammation and bleeding [62]. Leukemia may cause gingival enlargement secondary to leukemic infiltration and reactive inflammation [63]. The absence of hematologic abnormalities and unremarkable radiographic findings effectively excluded leukemia and plasma cell gingivitis from consideration. EGPA was included in the differential due to the presence of peripheral eosinophilia and its known association with hyperplastic gingivitis in 6%–13% of cases, with oral lesions occasionally representing the initial disease manifestation in approximately 2% of patients [64]. However, the lack of systemic features such as pulmonary or neurological involvement rendered EGPA unlikely [65]. PG, a common pregnancy‐associated lesion of the oral cavity, was also considered as it typically presents as a solitary, red, pedunculated papule with a friable surface [62].

Histopathology is crucial for diagnosing clinically nonspecific lesions [66]. The initial biopsy was limited and lacked the hallmark capillary proliferation with epithelioid endothelial cells; moreover, the prominent lympho‐eosinophilic infiltrate resembled common inflammatory lesions, contributing to a nondiagnostic result. In the second assessment, classic ALHE features were evident, including vascular proliferation with epithelioid endothelial cells, plasma cells, and lymphoid follicles [11]. Differentiation from epithelioid hemangioendothelioma and epithelioid angiosarcoma was supported by the absence of stromal cords, pleomorphism, mitoses, or necrosis [50, 51]. Although not applied here, nuclear FOSB staining could further help distinguish ALHE from similar malignant vascular tumors in difficult cases [67].

ALHE must also be distinguished from Kimura disease (KD) and Castleman disease (CD). KD features lymphadenopathy, eosinophilia, and elevated IgE, with histology showing germinal centers and eosinophilic microabscesses [5]. Unlike ALHE, KD lacks histiocytoid endothelial cells [68]. CD presents with systemic symptoms and lacks eosinophilia [69, 70]. The absence of systemic involvement ruled out KD and CD. Table S1 summarizes the histopathological differences among ALHE and other vascular/reactive lesions that are more commonly encountered in the oral cavity.

A potential link between ALHE and sex hormones has been proposed (Table 2), supported by pregnancy‐related exacerbation, postpartum regression, and reports of improvement following hormonal contraceptive withdrawal [77]. Theofilou et al. [5] reported an ALHE case in a patient using contraceptives for 20 years, suggesting estrogen‐mediated endothelial changes. The present case supports this hypothesis, as lesion regression followed contraceptive discontinuation. This hormonal responsiveness parallels that of PG, which is also frequently associated with pregnancy and oral contraceptive use, particularly in oral mucosal sites. Despite distinct histopathologic criteria separating ALHE from PG, the shared association with hyperestrogenic states, antecedent trauma, and Wilms tumor 1 (WT1) immunoreactivity suggests a possible convergence in early pathogenic mechanisms [1]. Furthermore, the discovery of a somatic TEK gene mutation, encoding the endothelial‐specific Tie‐2 receptor, in a case of dermal ALHE implicates aberrant angiogenic signaling pathways similar to those involved in other acquired vascular anomalies, including PG [71]. These findings raise the possibility of a molecularly convergent yet phenotypically divergent spectrum of reactive and neoplastic vascular proliferations, influenced by hormonal, immunologic, and genetic factors.

**TABLE 2:** ALHE cases associated with pregnancy and/or contraceptives.

References	Cases	Age (years)	Pregnancy/contraceptive	Location of lesions	Lesions (single or multiple)	Occurrence/worsening/regression after delivery or contraceptive discontinuation	Treatment
Olsen and Helwig [67]	5	—	Pregnancy	Not available	Single, multiple	Occurrence, regression	Not mentioned
Moy et al. [71]	2	28; 28	Pregnancy; contraceptive	Forehead and scalp	Multiple	Occurrence, worsening, regression	Electrodesiccation; curettage, laser/surgical excision
Hollo et al. [72]	1	28	Pregnancy	Eyebrow and temporal region	Multiple	Occurrence	Surgical excision
Zarrin‐Khameh et al. [73]	1	33	Pregnancy	Ear	Single	Occurrence	Surgical excision
Ceyhan et al. [74]	1	33	Pregnancy	Ear	Single	Occurrence	Surgical excision
Marcum et al. [75]	1	29	Pregnancy	Scalp	Multiple	Worsening and occurrence of new lesions	Laser
Parimalam and Thomas [76]	1	33	Pregnancy; contraceptive	Ear	Multiple	Occurrence	Surgical excision
Haritha et al. [77]	3	27; 25; 32	Pregnancy	Postauricular, scalp	Multiple	Occurrence, worsening, regression	Surgical excision, cryotherapy
Damarla et al. [78]	1	28	Pregnancy	Left upper extremity, axilla and trunk	Multiple	Persistent 8 months after delivery	NR
Theofilou et al. [5]	1	37	Contraceptive	Forehead	Single	Occurrence	Surgical excision
Cenk et al. [4]	1	28	Pregnancy	Ear	Multiple	Persistent 1.5 months after delivery^a^	Corticosteroid injection and topical, laser
Current case	1	30	Contraceptive	Gingiva	Single	Occurrence, regression	Corticosteroid injection

^a^ Patient presented with multiple lesions during each of her three pregnancies, which regressed spontaneously after delivery in the first two pregnancies but have persisted since the third pregnancy.

The relationship between epithelioid hemangioma (EH) and ALHE has long been debated due to overlapping histological features [11, 79, 80, 81]. Emerging evidence, however, supports a key distinction: EH, particularly in bone and soft tissue, represents a true neoplasm frequently driven by FOS/FOSB fusions, while ALHE appears to be a reactive inflammatory process lacking these genetic alterations [79, 80, 81]. Clinically, this distinction is relevant, as EH requires careful follow‐up given its neoplastic behavior [80], whereas ALHE typically follows an indolent course and may respond to conservative or anti‐inflammatory management. This molecular insight has reshaped diagnostic criteria and emphasized the need for morpho‐molecular integration in vascular tumor classification.

The standard treatment for oral ALHE is complete surgical excision, though corticosteroids (topical, systemic, or intralesional) offer viable alternatives [17]. In this case, a conservative approach was adopted due to significant clinical improvement following contraceptive discontinuation and subgingival scaling. Intralesional triamcinolone acetonide was administered for its anti‐inflammatory and angiogenesis‐inhibiting effects [82]. The absence of recurrence over 3 years suggests that conservative management may be an effective alternative to surgery.

In conclusion, this case is of particular interest due to the unusual intraoral presentation of ALHE, a condition classically described in cutaneous sites, but rarely reported in the gingiva. The lesion's clinical appearance mimicked inflammatory and granulomatous diseases, and initial histopathological findings were non‐specific, underscoring the diagnostic complexity. Notably, the lesion demonstrated clinical regression following hormonal withdrawal and conservative periodontal therapy, with complete resolution achieved through a single intralesional corticosteroid injection. The identification of cutaneous‐type histopathologic features, such as epithelioid endothelial cells and eosinophilic infiltrates, further reinforces the relevance of this case for specialists in mucocutaneous pathology. This report not only expands the clinicopathologic spectrum of ALHE but also supports a conservative approach in specific oral presentations.

## Author Contributions

**Pedro Vinícius Santos de Jesus:** investigation, writing – original draft; **Louise Cristina Santos:** investigation. **John Lennon Silva Cunha:** conceptualization, methodology. **Gilberth Tadeu Dos Santos Aciole:** conceptualization, methodology. **Nicole Lonni:** writing – review and editing; **Matheus Antoni da Silva Costa:** writing – review and editing. **Rogério Gondak:** validation. **Elena Riet Correa Rivero:** validation. **Ricardo Luíz Cavalcanti de Albuquerque‐Júnior:** project administration, supervision, funding acquisition, writing – review and editing. All authors approved the version to be published and agreed to be accountable for all aspects of the work, ensuring that questions related to the accuracy or integrity of any part of the work are appropriately investigated and resolved.

## Funding

This work was supported by Coordenação de Aperfeiçoamento de Pessoal de Nível Superior, 001 and Conselho Nacional de Desenvolvimento Científico e Tecnológico, 1231650120376438.

## Ethics Statement

This study was conducted in accordance with the ethical standards of the institutional and national research committee and with the 1964 Helsinki Declaration and its later amendments. Ethical approval for this case report was obtained from the Institutional Review Board under protocol number 25000.237810/2014‐54. Written informed consent was obtained from the patient for the publication of this case and accompanying images.

## Consent

Informed consent was obtained from all individual participants included in the study.

## Conflicts of Interest

The authors declare no conflicts of interest.

## Supporting information


**Table S1:** Comparison of histopathological features between ALHE and possible differential diagnosis.

## Data Availability

Data sharing not applicable to this article as no datasets were generated or analysed during the current study.
