# Supplementary material for: Oral Angiolymphoid Hyperplasia With Eosinophilia Exhibiting Cutaneous‐Type Histopathologic Features: Clinical Regression Following Hormonal Withdrawal and a 50‐Year Review
Source: J Cutan Pathol. 2026 Feb 24;53(6):491–501. doi: 10.1111/cup.70072 (PMC13136054; doi:10.1111/cup.70072)
Supplement: Supplementary file 1 — Table S1: Comparison of histopathological features between ALHE and possible differential diagnosis. [file CUP-53-491-s001.docx]

**Supplementary Material**

**Supplementary Table 1.** Comparison of histopathological features between ALHE and possible differential diagnosis.

| Features | ALHE | Kimura Disease | Castleman disease | Haemangioma | Pyogenic Granuloma | Eosinophilic granulomatosis with polyangiitis |
| --- | --- | --- | --- | --- | --- | --- |
| Depth | Dermis, subcutaneous | Subcutaneous, musculature | Dermis, Subcutaneous, lymph nodes | Dermis, Subcutaneous | Dermis,  Subcutaneous | Deep dermis, subcutaneous tissue, vessels |
| Infiltrate | More diffuse | Nodular | More diffuse | Minimal or absent | Mild, often perivascular | Dense, mixed |
| Germinating center | Uncommon | Always | Common | Absent | Absent | Absent |
| Eosinophilia | Slightly evident | Massive | Rare | Rare | Absent or minimal | Massive |
| Neoangiogenesis | Prominent | Less prominent | Prominent | Prominent | Prominent | Minimal |
| Epithelioid endothelium | Present | Absent | Present | Absent | Absent | Absent |
| Lymphoid follicle | Rare | Common | Common | Absent | Absent | Rare |
| Eosinophilic abscesses | Not seen | Present | Not seen | Not seen | Not seen | Present |
| Fibrosis | Slight | Commonly evident | Common | May be present | Often absent | May be present |
| Edema | Minimal | Often evident | Present | Variable | Present | Present |

Supplementary References:

S1. Brahs A, Sledge B, Mullen H, Newman A, Mengesha Y, Estrada S. Angiolymphoid Hyperplasia with Eosinophilia: Many Syllables, Many Unanswered Questions. J Clin Aesthet Dermatol. 2021;14(6):49-54.

S2. Botto E, Rodriguez-Waitkus P, Albers SE. Angiolymphoid hyperplasia with eosinophilia and Kimura disease: A case report and literature review. Pediatr Dermatol. 2024;41(5):857-860.

S3. Srivastava H, Reddy DS, Shah SN, Shah V. Castleman’s disease. J Oral Maxillofac Pathol. 2020;24(3):593.

S4. Emmi G, Bettiol A, Gelain E, et al. Evidence-Based Guideline for the diagnosis and management of eosinophilic granulomatosis with polyangiitis. Nat Rev Rheumatol. 2023;19(6):378-393.

S5. Olsen TG, Helwig EB. Angiolymphoid hyperplasia with eosinophilia. A clinicopathologic study of 116 patients. J Am Acad Dermatol. 1985;12(5 Pt 1):781-796.
